# Cherry Pollen and Bee Bread Enhance Fitness of *Chaetodactylus hirashimai* (Acari: Sarcoptiformes): A Two-Sex Life-Table Evaluation of Four Pollen Diets

**DOI:** 10.3390/insects17080797

**Published:** 2026-07-31

**Authors:** Zhaoyun Lyu, Hongyan Song, Yue Zhang, Qitong Huang, Meng Sun

**Affiliations:** Shandong Institute of Sericulture, Shandong Academy of Agricultural Sciences, Yantai 265503, China; lzhy67188@sina.com (Z.L.); songhy_77@126.com (H.S.); zzy15927001455@163.com (Y.Z.); 13760637249hqt@nwafu.edu.cn (Q.H.)

**Keywords:** *Chaetodactylus hirashimai*, *Osmia excavata*, cherry pollen, bee bread, two-sex life table

## Abstract

The mason bee *Osmia excavata* Alfken (Hymenoptera: Megachilidae) is an essential pollinator for rosaceous fruit trees in northern China, but its populations are declining because of severe infestations by the mite *Chaetodactylus hirashimai* Kurosa (Acari: Sarcoptiformes). This mite feeds on pollen within mason bee nest tubes, thereby impairing larval development. However, the effects of different floral resources on the fitness of *C. hirashimai* remain unclear. Herein, we evaluated the development, survival, and reproduction of *C. hirashimai* reared on four diets (cherry pollen, cherry bee bread, apple pollen, or apple bee bread), by using an age-stage, two-sex life table. *Chaetodactylus hirashimai* successfully completed its life cycle on all diets. Cherry pollen supported shorter pre-adult development, longer adult longevity, and higher fecundity than apple pollen. Bee bread diets resulted in significantly lower pre-adult mortality (11.76–14.81%), and higher fecundity (270–278 eggs/female) and net reproductive rate (*R*_0_ = 115–120 offspring/individual), than raw pollen (44.64–44.83% mortality; 117–179 eggs/female; *R*_0_ = 40–50 offspring/individual). No significant differences in the intrinsic rate of increase (*r*) and finite rate of increase (*λ*) were observed among diets. Our findings demonstrated that cherry pollen is a highly favorable resource for *C. hirashimai*, and bee bread further enhances mite population growth. This study highlights the ecological conflict between cherry pollination and mite infestation, and these life-table results can provide preliminary laboratory support for subsequent research on integrated mite control in fruit orchards.

## Simple Summary

The mason bee *Osmia excavata* is a key pollinator of early-spring fruit trees in northern China, but its populations are severely threatened by the mite *Chaetodactylus hirashimai*. This mite feeds on pollen inside mason bee nests, thus decreasing bee survival and pollination efficiency. Here, using two-sex life tables, we investigated how cherry pollen, cherry bee bread, apple pollen, and apple bee bread affect *C. hirashimai* development, survival, and reproduction. Cherry pollen strongly promoted mite development and reproduction, whereas bee bread, compared with raw pollen, significantly improved mite fitness. These findings highlight that cherry-dominated orchards might exacerbate mite infestations, thus posing a major risk to mason bee health. Our study provides critical information for developing sustainable pest management strategies that balance pollinator conservation and mite control in orchard ecosystems.

## 1. Introduction

The orchard mason bee, *Osmia excavata* Alfken (Hymenoptera: Megachilidae), is a critical pollinator of rosaceous fruit trees in northern China [1,2,3]. Because of its low developmental threshold temperature, high foraging frequency, and superior pollination efficiency, it has been widely used for the pollination of economically important fruit trees, such as apple, pear, and peach [4,5]. Pollination by *O. excavata* significantly improves fruit set and quality, and outperforms both honeybee pollination and artificial pollination [6,7]. This species is therefore recognized as an important resource insect in China [8,9,10]. However, wild and artificially reared populations of *O. excavata* have markedly declined in recent years, and infestation by nest-inhabiting mites has become a key factor limiting population expansion and field application [11,12,13,14,15].

*Chaetodactylus hirashimai* Kurosa (Acari: Sarcoptiformes) is a major mite pest of *Osmia excavata*. This species was originally described and native to Japan; it was first discovered and documented in China in 2016, and has since dispersed widely in northern fruit orchards and severely harmed local mason bee populations [16,17]. This mite has two different morphs: the reproductive type and the dispersal type. The reproductive morphs are the primary form colonizing brood cell pollen masses; they feed heavily on stored pollen, reproduce prolifically, and leave abundant brown powdery residues inside nest cavities. This heavy exploitation of pollen deprives mason bee larvae of food resources, leading to larval developmental retardation or mortality [17,18]. Ultimately, survival rates across all mason bee life stages inside nest tubes drop substantially, and adult emergence rates in the subsequent spring are severely reduced [19]. The severe decrease in mason bee reproductive success subsequently impairs pollination services. Studies on the closely related species *Osmia cornifrons* (Radoszkowski, 1887) (Hymenoptera: Megachilidae) have confirmed that pollen resource exploitation by *Chaetodactylus* spp. and *Tortonia* sp. is the main cause of host population decline, and mite abundance is closely associated with nest microclimate and food resources [20,21]. Although artificial mite removal and safety assessments of acaricides have been reported to reduce mite infestation, systematic studies on the effects of plant-derived pollen resources on the reproductive fitness of *C. hirashimai* remain limited [22,23].

Pollen, the primary nutrient resource within mason bee nests, serves not only as the basis for larval development but also as a key food source for nest-associated symbionts and parasites. Pollen from different plant species substantially differs in nutritional composition, secondary metabolite content, and physical traits, which together can directly affect mite development and reproductive potential [24,25]. Cherry and apple, major early-spring nectar and pollen sources in northern orchards, have distinct pollen quality [26]. Furthermore, bee bread, formed from the fermentation of collected pollen mixed with glandular secretions by mason bees, has markedly different physicochemical properties from raw pollen and may exert divergent ecological effects on mites [27]. Currently, a lack of systematic life-table studies on reproductive *C. hirashimai* fed different pollen and bee bread diets hinders comprehensive understanding of the tripartite interactions among pollen plants, mason bees, and pest mites.

Herein, we used an age-stage, two-sex life-table to systematically evaluate the effects of four diets (cherry pollen, cherry bee bread, apple pollen, and apple bee bread) on the development, survival, and reproduction of *C. hirashimai*. This study was aimed at clarifying the regulatory effects of different pollen resources on mite population fitness; elucidating the mechanisms underlying the effects of bee bread versus raw pollen on mite fitness; and revealing the potential ecological conflict between cherry resources and mite outbreaks. Our findings provide a scientific basis for the sustainable conservation of *O. excavata* and integrated management of pest mites through pollen plant management in orchard ecosystems.

## 2. Materials and Methods

### 2.1. Rearing and Feed Treatments

A colony of *C. hirashimai* was collected from a mason bee house in an orchard (N37.2458°, E121.1743°) in Yantai, Shandong Province, China. The culture was kept under controlled conditions (27 ± 2 °C; 65 ± 5% RH) in the dark with mixed pollen. The mixed pollen sample consisted of three types (apricot, camellia, and oilseed rape), each composing one-third of the total mixture. These pollens were collected with traps placed in honeybee hives in Zhejiang Province, China. Microscopic analysis of the pollen composition revealed that *Camellia* pollen was dominated by *Camellia* sp. (89%), with smaller amounts of *Brassica* sp. (5%), and only traces of *Acer* sp., *Armeniaca* sp., and *Lactuca* sp. In contrast, apricot pollen consisted primarily of *Armeniaca* sp. (68%), followed by *Brassica* sp. (31%), plus trace levels of *Seriphidium* sp. Finally, oilseed rape pollen comprised mostly *Brassica* sp. (76%), along with *Armeniaca* sp. (10%) and *Salix* sp. (9%), and minor contributions from *Seriphidium* sp. (4%).

Four feed treatments were examined: cherry pollen, cherry bee bread, apple pollen, and apple bee bread. The preparation methods for cherry pollen and apple pollen followed a protocol described by Liu and Li [28]. Briefly, flower buds in the late budding stage, or newly opened cherry and apple flowers, were collected and immediately transported to the laboratory. Anthers were manually dissected and placed in a constant-temperature drying oven at 25 °C. After anther dehiscence and natural pollen release, the collected pollen was immediately transferred to a −18 °C refrigerator for subsequent storage and use. For the preparation of cherry bee bread and apple bee bread, during the cherry and apple flowering period, *Osmia excavata* were released in cherry and apple orchards, respectively, to forage for pollen and nectar. Sealed nest tubes were then collected and brought back to the laboratory, and bee bread was carefully extracted from the nest tubes. Pollen morphological identification was performed to screen qualified samples. Only cherry bee bread containing >95% cherry pollen and apple bee bread containing >95% apple pollen were selected and stored at −18 °C for subsequent experiments.

### 2.2. Life-Table Study

After each feed pre-treatment, female *C. hirashimai* were placed in Petri dishes with ample feed and allowed to lay eggs freely for 24 h. Sufficient eggs were collected (50 ± 10) and placed in a plastic dish (20.0 mm in diameter and 10.0 mm in depth). Newly hatched larvae were transferred individually into plastic dishes (20.0 mm diameter, 10.0 mm depth) and provided with sufficient diet daily. In total, 52 eggs were supplied with cherry pollen; 51 eggs were supplied with cherry bee bread; 52 eggs were supplied with apple pollen; and 54 eggs were supplied with apple bee bread. After the deutonymph had developed into adults, the newly emerged adults were paired in a 1:1 female: male ratio in a new plastic dish. All experimental individual development, survival/longevity, and fecundity were recorded daily until death. All life-table experiments were performed under laboratory conditions at 27 ± 2 °C; 65 ± 5% RH, and a 0:24 h (L:D) photoperiod.

### 2.3. Life-Table Analysis

The raw life-table data for all *C. hirashimai* individuals (developmental time, survivorship, and female daily fecundity) were analyzed on the basis of age-stage, two-sex life-table theory [29,30,31,32,33] in the computer program TWOSEX-MSChart (Ver. 5/7/2024) [34].

The age-stage-specific survival rate (*s_xj_*, where *x* = age, and *j* = stage); age-stage-specific fecundity (*f_x_*_j_); age-specific survival rate (*l_x_*); age-specific fecundity (*m_x_*); and population parameters including the intrinsic rate of increase (*r*), finite rate of increase (*λ*), net reproductive rate (*R*_0_), and mean generation time (*T*), were calculated according to Chi and Liu [29,35]. The bootstrap method was used to estimate the standard errors of the developmental time, fecundity, longevity, and population parameters with 100,000 bootstraps. The results of treatments were compared with the paired bootstrap test according to the confidence interval of the difference [36].

## 3. Results

### 3.1. Developmental Time, Longevity, and Fecundity

When provided with the four tested feeds, *C. hirashimai* was able to complete its life cycle and reproduce successfully. The developmental times of total preadult stages reared on apple bee bread were significantly longer than those reared on cherry bee bread, apple pollen, or cherry pollen (*p* < 0.05), whereas no significant difference was observed between *C. hirashimai* reared on apple pollen versus cherry pollen (*p* = 0.95479) (Table 1).

The preadult mortality of the individuals fed on cherry bee bread (11.76%) was significantly lower than that of the ones fed on cherry pollen (44.83%) (*p* = 0.00004; Table 2). Similarly, the preadult mortality of *C. hirashimai* fed on apple bee bread (14.81%) was significantly lower than that of *C. hirashimai* fed on apple pollen (44.64%) (*p* = 0.00027). In contrast, no significant difference in preadult mortality was observed between pollen groups (*p* = 0.95015) or between bee bread groups (*p* = 0.66639).

**Figure 1:**
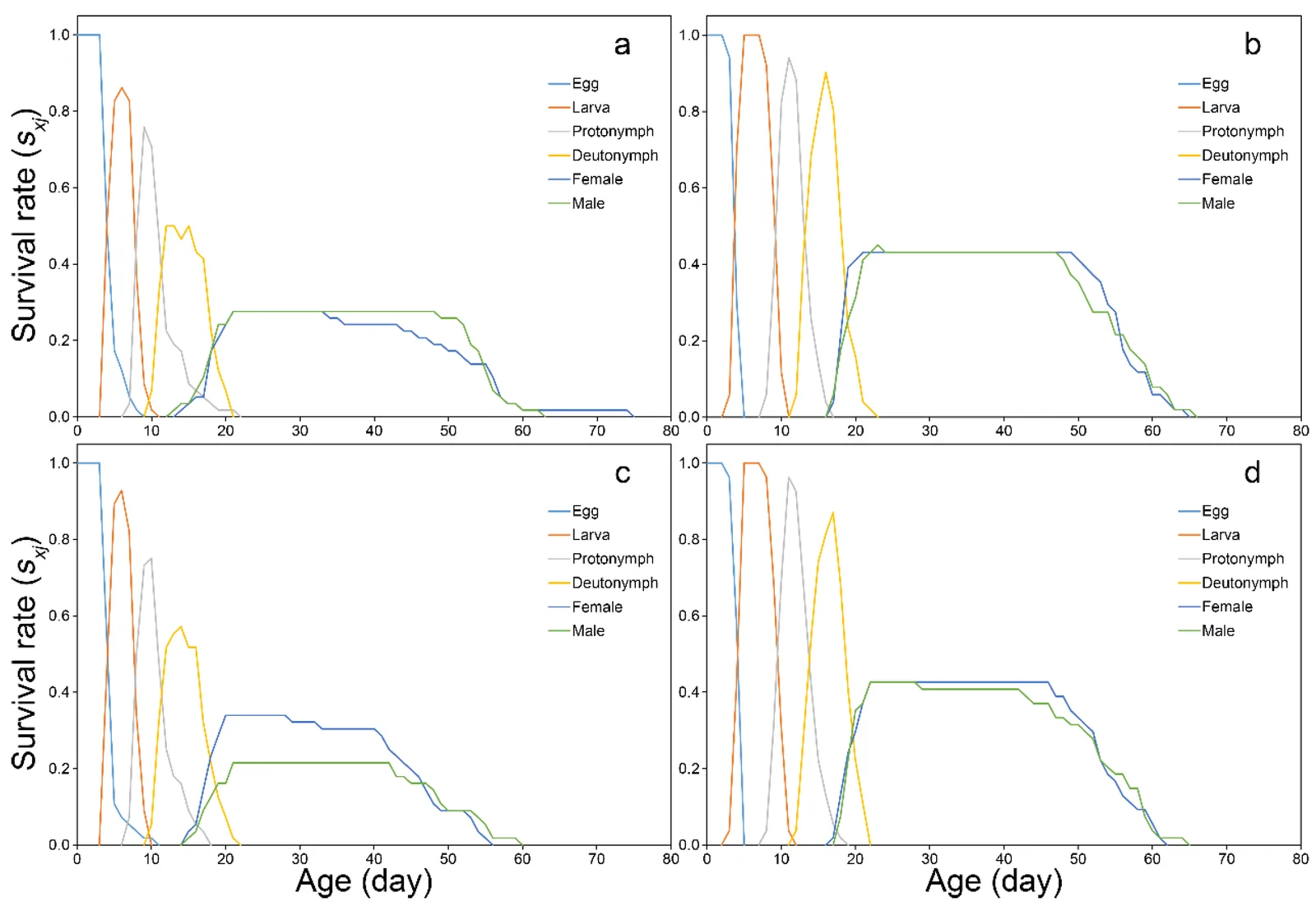
Age-stage-specific survival rates (*s_xj_*) of *Chaetodactylus hirashimai.* (**a**) *C. hirashimai* reared on cherry pollen. (**b**) *C. hirashimai* reared on cherry bee bread. (**c**) *C. hirashimai* reared on apple pollen. (**d**) *C. hirashimai* reared on apple bee bread.

A comparison of the age-stage survival rates (*s_xj_*) of *C. hirashimai* reared on cherry pollen, cherry bee bread, apple pollen, or apple bee bread (Figure 1) indicated clear overlap between the end of each life stage and the start of the next stage.

The average adult longevity of *C. hirashimai* reared on apple pollen (29.90 days) was significantly shorter than that of *C. hirashimai* reared on cherry pollen (35.94 days, *p* = 0.00074), cherry bee bread (36.62 days, *p* < 0.00001), or apple bee bread (34.28 days, *p* = 0.00243). The mean fecundity of *C. hirashimai* reared on cherry bee bread (278.55 eggs/female) or apple bee bread (270.65 eggs/female) was significantly higher than that of *C. hirashimai* reared on cherry pollen (179.50 eggs/female) or apple pollen (117.05 eggs/female) (*p* < 0.0001). No significant difference in fecundity was detected between mites fed on cherry bee bread versus apple bee bread (*p* = 0.62545) (Table 3).

**Figure 2:**
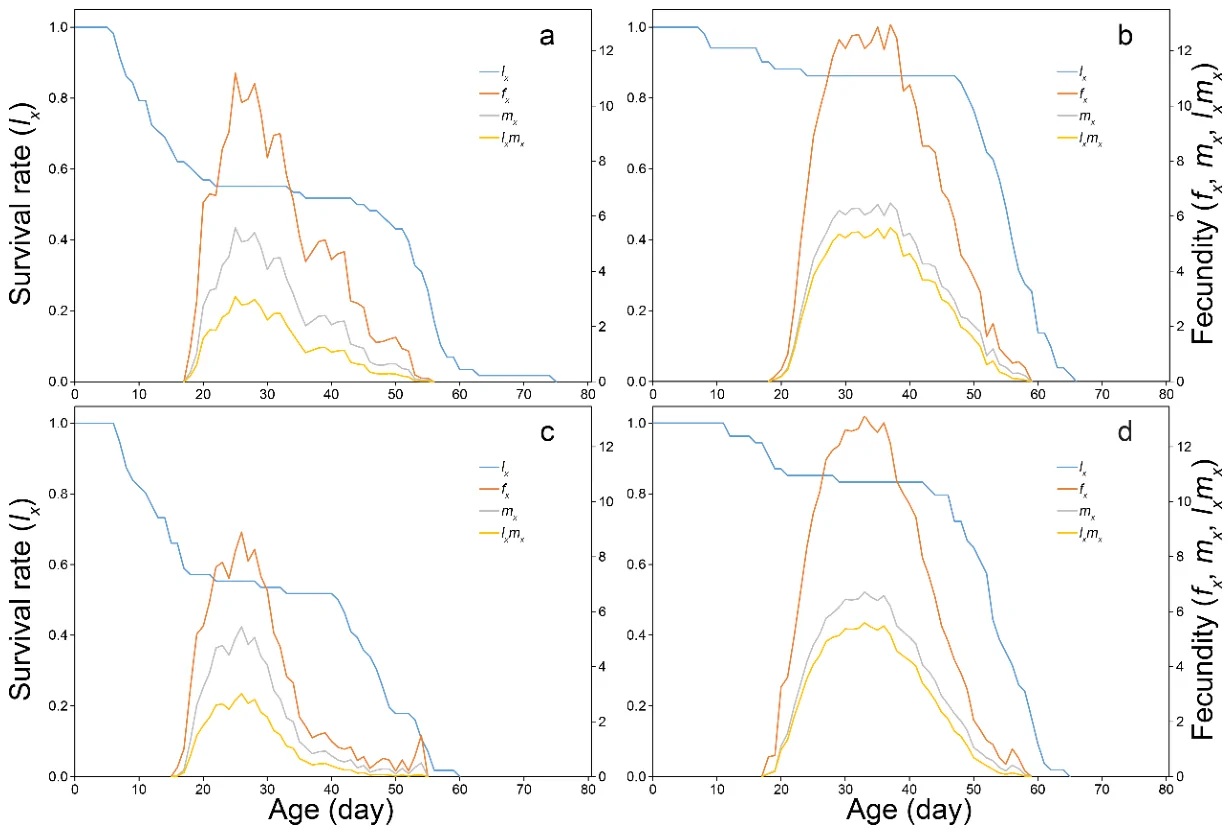
Age-specific survival rate (*l_x_*), age-stage-specific fecundity (*f_x_*), age-specific fecundity (*m_x_*), and maternity (*l_x_m_x_*) of *Chaetodactylus hirashimai.* (**a**) *C. hirashimai* reared on cherry pollen. (**b**) *C. hirashimai* reared on cherry bee bread. (**c**) *C. hirashimai* reared on apple pollen. (**d**) *C. hirashimai* reared on apple bee bread.

The *l_x_* decreased with increasing age, whereas *f_x_*, *m_x_*, and *l_x_m_x_* initially increased but subsequently decreased with age under each treatment (Figure 2).

**Figure 3:**
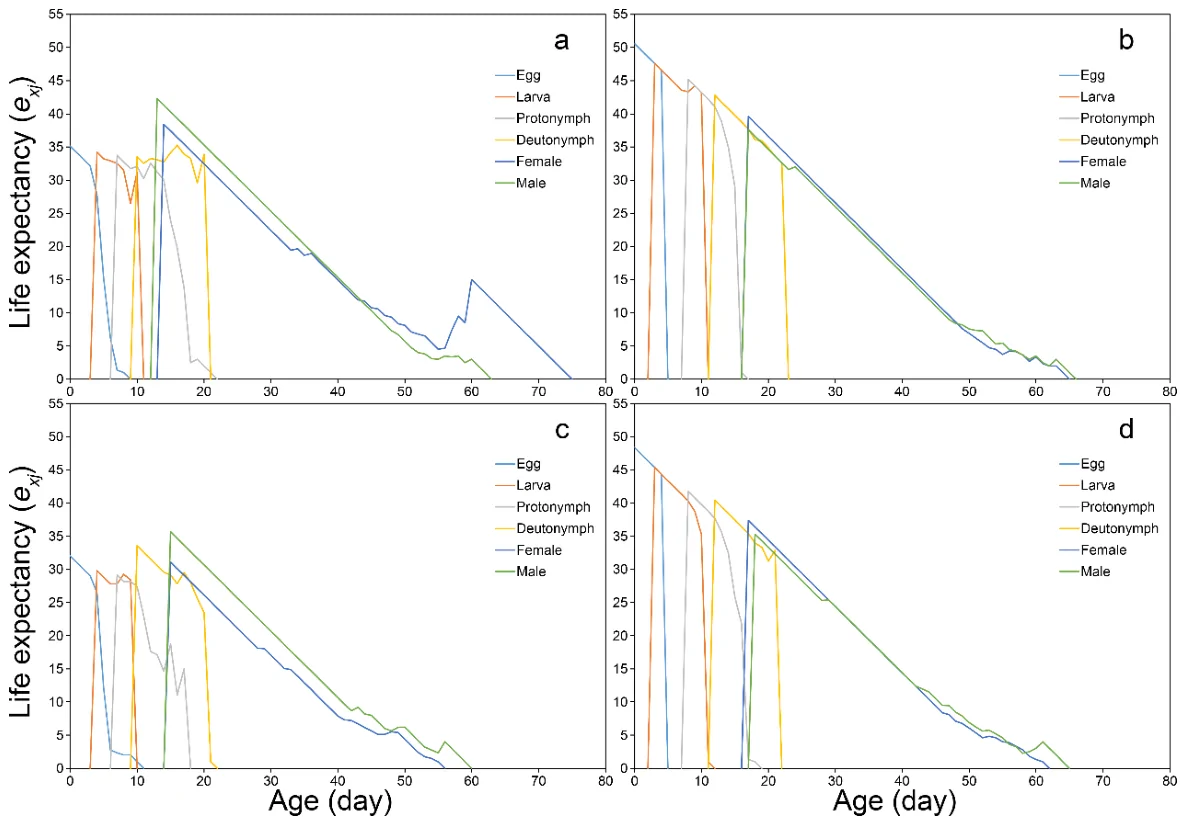
Life expectancy (*e_xj_*) of *Chaetodactylus hirashimai.* (**a**) *C. hirashimai* reared on cherry pollen. (**b**) *C. hirashimai* reared on cherry bee bread. (**c**) *C. hirashimai* reared on apple pollen. (**d**) *C. hirashimai* reared on apple bee bread.

The life expectancy (*e_xj_*) of *C. hirashimai* reared on the four diets gradually decreased with increasing age. Males and females fed on the two bee bread diets exhibited greater life expectancy than those reared on the two pollen diets (Figure 3).

**Figure 4:**
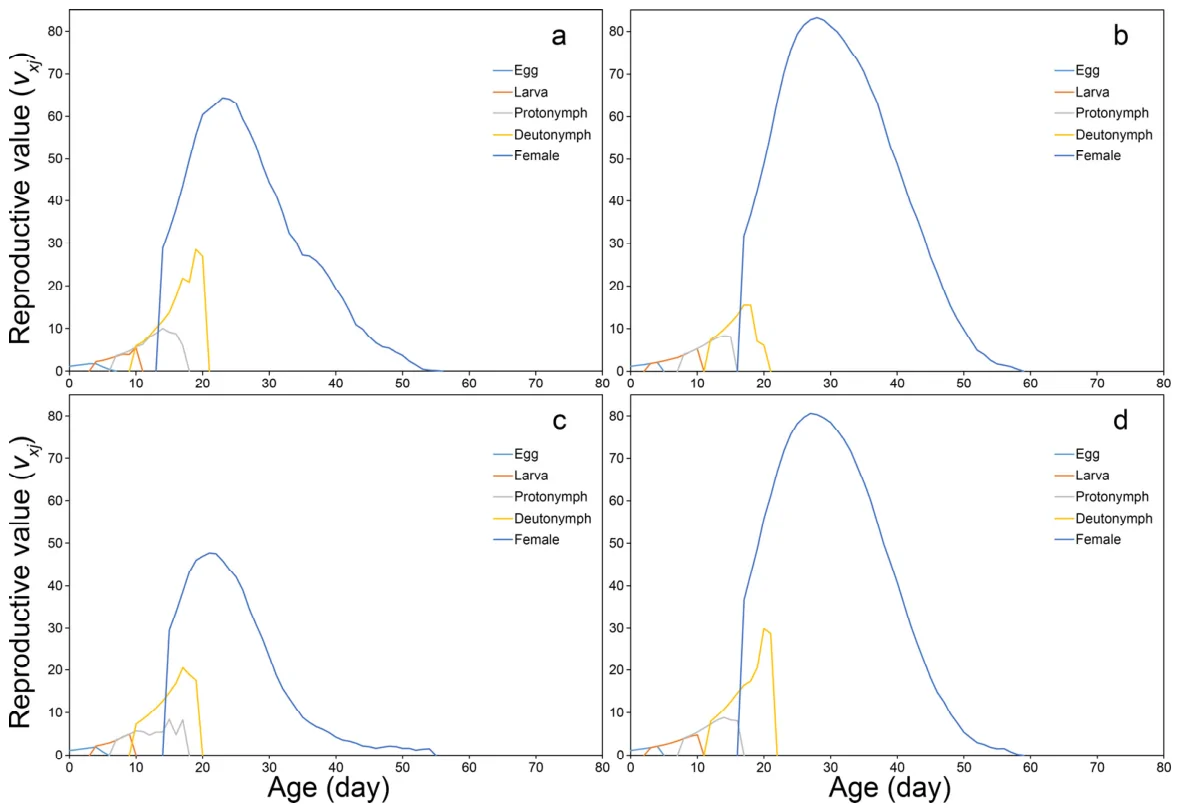
Reproductive value (*v_xj_*) of each age-stage of *Chaetodactylus hirashimai.* (**a**) *C. hirashimai* reared on cherry pollen. (**b**) *C. hirashimai* reared on cherry bee bread. (**c**) *C. hirashimai* reared on apple pollen. (**d**) *C. hirashimai* reared on apple bee bread.

The reproductive value (*v_xj_*) of *C. hirashimai* peaked at different ages depending on the diet: 64.43 at day 23 (cherry pollen), 83.23 at day 28 (cherry bee bread), 47.71 at day 21 (apple pollen), and 80.62 at day 27 (apple bee bread) (Figure 4).

### 3.2. Life-Table and Population Parameters

No significant differences were observed in *r* and *λ* among *C. hirashimai* reared on the four tested diets (*p* > 0.1) (Table 4). The *R*_0_ of *C. hirashimai* fed on the two bee bread diets was significantly higher than that of mites reared on the two pollen diets (*p* < 0.01). The T of *C. hirashimai* was as follows, in descending order: cherry bee bread > apple bee bread > cherry pollen > apple pollen.

## 4. Discussion

This study systematically evaluated the effects of cherry pollen, cherry bee bread, apple pollen, and apple bee bread on the fitness of reproductive *C. hirashimai*, a newly recorded kleptoparasitic mite associated with the orchard mason bee *Osmia excavata* in northern China. The two-sex life-table results show that *C. hirashimai* exhibits superior developmental and reproductive performance when fed raw cherry pollen compared with raw apple pollen. Meanwhile, bee bread from both cherry and apple sources substantially improves mite fitness relative to unprocessed raw pollen of the same plant origin. These findings not only reveal the nutritional adaptation of *C. hirashimai* to rosaceous pollen resources but also highlight a critical ecological conflict between cherry pollination services and mite population outbreaks, with important implications for the sustainable management of mason bees and pest mites in orchard ecosystems.

Pollen quality is a key determinant of the performance of pollen-feeding arthropods, including both pollinators and their natural enemies or kleptoparasites [37]. Moreover, life-table analysis has become a powerful tool to quantify these effects under controlled conditions [38]. Herein, we observed that *C. hirashimai* exhibited better development and reproduction on cherry pollen than on apple pollen, thus indicating that cherry pollen is a nutritionally superior resource for this mite. However, targeted chemical analyses of pollen nutrients and secondary metabolites were not performed in the present study to verify this inference. This difference might be attributable to variations in nutritional composition between cherry and apple pollen, because host plant quality directly shapes mite developmental trajectories and reproductive potential [39]. Cherry pollen generally contains higher levels of crude protein, essential amino acids, and soluble sugars than apple pollen, and therefore can directly support faster larval growth, longer adult longevity, and higher fecundity in mites [40]. In addition, secondary metabolites such as flavonoids and phenolics in pollen might also influence mite performance and cherry pollen might contain lower levels of defensive compounds that inhibit mite development and therefore might be more suitable for *C. hirashimai* [41]. As a dominant early-spring floral resource in northern China, cherry pollen is the primary food for *O. excavata* during its active period. Our findings therefore suggest a critical concern: cherry-dominated orchard landscapes, while supporting mason bee pollination, simultaneously could favor the proliferation of *C. hirashimai*, thus posing a hidden risk to mason bee populations.

Bee bread, a bee-processed pollen product, is nutritionally richer and more digestible than raw pollen, due to microbial fermentation, the addition of glandular secretions, and partial degradation of complex polysaccharides and proteins [42,43]. Our study confirmed that bee bread diets markedly improved *C. hirashimai* survival and reproduction, by decreasing pre-adult mortality by ~70% and increasing fecundity by 1.5–2.3 times that observed with raw pollen. The significantly elevated *R*_0_ on bee bread further indicated that this high-quality resource strongly drives mite population growth. In mason bee nests, because bee bread is the exclusive food of developing larvae, *C. hirashimai* can easily access this high-quality resource. This aspect creates a paradox in which the food that nurtures the next generation of mason bees also provides an ideal medium for the reproduction of their kleptoparasitic mites. Mites and mason bee larvae rely on the same pollen food resource and therefore compete intensely for limited nutrition, which poses a major threat to the survival of mason bees.

Notably, although both cherry and apple bee bread significantly enhanced mite fitness, no significant differences in fecundity or *R_0_* were observed between bee bread types. Therefore, the bee-processing effect overrode differences in pollen quality, thereby homogenizing the nutritional value of different pollens into a highly suitable resource for *C. hirashimai*. Therefore, even if apple pollen is less favorable for mites, it can still support robust mite reproduction after being processed into bee bread by mason bees. Consequently, mite infestation risks may occur in both cherry and apple orchards, yet cherry orchards potentially face higher baseline risk, which could be attributed to the relatively superior nutritional quality of raw cherry pollen.

From an ecological perspective, this study revealed a complex tripartite interaction among pollen plants, mason bees, and kleptoparasitic mites. Cherry trees rely on *O. excavata* for efficient pollination and fruit production; *O. excavata* depends on cherry pollen for its own survival and reproduction; and *C. hirashimai* exploits both raw pollen and bee bread within the nest. This interdependence creates a delicate balance that could easily be disrupted by changes in floral resource composition or mite abundance. In recent decades, the expansion of cherry cultivation in northern China has increased the availability of cherry pollen, which, as shown herein, directly benefits *C. hirashimai*. This aspect might partially explain the increasing prevalence of mite infestations in commercial mason bee populations in cherry-growing regions.

From an applied perspective, our findings provide actionable insights for the integrated management of *C. hirashimai* and the conservation of *O. excavata*. Current mite control methods, such as chemical acaricides and nest disinfection, are either harmful to mason bees or ineffective against mites hidden inside nest tubes [2]. Our results imply that adjusting orchard floral resource composition could serve as a promising ecological control approach worthy of further exploration. For instance, interplanting non-rosaceous flowering plants that are less favorable to *C. hirashimai* within cherry orchards may dilute the abundance of cherry pollen and thereby lower mite fitness; however, additional follow-up research is required to verify how this mite responds to alternative pollen sources. Additionally, developing mite-resistant pollen varieties or microbial additives that inhibit mite development without affecting mason bee health could be explored.

This study has several limitations that warrant future research. First, because we tested only two common rosaceous pollens, future studies should include a broader range of plant species to better understand the host plant range of *C. hirashimai*. Second, we focused on the direct effects of pollen and bee bread on mite fitness; however, the underlying molecular and physiological mechanisms, such as nutrient absorption and immune response, remain unclear and require further investigation. Third, because the experiment was conducted under controlled laboratory conditions, field trials are needed to validate the findings in real orchard environments, where factors such as temperature, humidity, and natural enemies might influence mite population dynamics.

In conclusion, this study provides the first systematic evidence, to our knowledge, that cherry pollen and bee bread are key drivers of *C. hirashimai* population growth in northern China. Our results advance understanding of the nutritional ecology of this newly recorded mite, as well as the tripartite interactions among nectar plants, mason bees, and kleptoparasitic mites. The identified ecological conflict between cherry pollination and mite infestation highlights the urgent need for sustainable management strategies that balance pollinator conservation and mite control in early-spring orchard ecosystems.

## 5. Conclusions

The mite *C*. *hirashimai* can complete its full life cycle when fed cherry pollen, cherry bee bread, apple pollen and apple bee bread, demonstrating that this mite can utilize a wide range of pollen resources derived from Rosaceae plants.

Compared with apple pollen, cherry pollen contributes to improved developmental performance, longer adult longevity and higher fecundity of mites, indicating cherry pollen is a relatively more suitable food resource for *C. hirashimai*. Across all tested diets, bee bread supports better overall mite fitness than raw pollen, which is reflected in reduced pre-adult mortality, extended adult lifespan, as well as substantially elevated fecundity and net reproductive rate (*R*_0_). No significant differences in intrinsic growth rate (*r*) or finite rate of increase (*λ*) were observed among the four diet groups, yet bee bread substantially elevates mite population expansion capacity by increasing (*R*_0_). Given the superior nutritional suitability of cherry pollen for mites, orchard landscapes dominated by cherry vegetation have the potential to facilitate population growth of *C. hirashimai*, which may create adverse impacts on the survival and pollination functions of the mason bee *O. excavata*.

This study systematically characterizes the performance of *C. hirashimai* fed different rosaceous pollen and bee bread resources, offering fundamental empirical evidence for understanding the nutritional ecology of this pest. Our findings also lay a theoretical foundation for developing targeted integrated pest management solutions, which can coordinate the dual goals of wild pollinator protection and mite population suppression in early-spring fruit orchards.

## Figures and Tables

**Table 1:** Development times of *Chaetodactylus hirashimai*.

Development Time	Feed Treatment							
	Cherry Pollen		Cherry Bee Bread		Apple Pollen		Apple Bee Bread	
	*n*	Mean ± SE	*n*	Mean ± SE	*n*	Mean ± SE	*n*	Mean ± SE
Egg duration (day)	52	4.52 ± 0.10 a	51	4.24 ± 0.08 b	52	4.54 ± 0.08 a	54	4.54 ± 0.08 a
Larva duration (day)	48	3.96 ± 0.12 b	48	5.46 ± 0.10 a	47	3.87 ± 0.10 b	52	5.38 ± 0.08 a
Protonymph duration (day)	41	3.85 ± 0.26 ab	46	4.15 ± 0.09 ab	36	3.69 ± 0.25 b	48	4.40 ± 0.11 a
Deutonymph duration (day)	32	5.59 ± 0.37 ab	45	5.13 ± 0.18 b	31	6.10 ± 0.29 a	46	5.26 ± 0.09 b
Preadult duration (day)	32	17.94 ± 0.36 c	45	18.96 ± 0.21 b	31	17.97 ± 0.30 c	46	19.57 ± 0.20 a

SEs were estimated with 100,000 bootstraps. Different letters in the same row represent significant differences with a paired bootstrap test (*p* < 0.05).

**Table 2:** Mortality of *Chaetodactylus hirashimai*.

Development Stage	Feed Treatment			
	Cherry Pollen	Cherry Bee Bread	Apple Pollen	Apple Bee Bread
Egg	10.34 ± 3.99% a	0.00 ± 0.00% b	7.14 ± 3.45% a	0.00 ± 0.00% b
Larva	6.90 ± 3.33% a	5.88 ± 3.30% a	8.93 ± 3.80% a	3.70 ± 2.56% a
Protonymph	12.07 ± 4.27% ab	3.92 ± 2.72% b	19.64 ± 5.31% a	7.41 ± 3.55% b
Deutonymph	15.52 ± 4.75% a	1.96 ± 1.95% b	8.93 ± 3.79% ab	3.70 ± 2.57% b
Preadult	44.83 ± 6.53% a	11.76 ± 4.50% b	44.64 ± 6.63% a	14.81 ± 4.83% b

SEs were estimated with 100,000 bootstraps. Different letters in the same row represent significant differences with a paired bootstrap test (*p* < 0.05).

**Table 3:** Longevity and fecundity of *Chaetodactylus hirashimai*.

Parameter	Feed Treatment							
	Cherry Pollen		Cherry Bee Bread		Apple Pollen		Apple Bee Bread	
	*n*	Mean ± SE	*n*	Mean ± SE	*n*	Mean ± SE	*n*	Mean ± SE
Adult longevity (day)	32	35.94 ± 1.33 a	45	36.62 ± 0.99 a	31	29.90 ± 1.16 b	46	34.28 ± 0.86 a
Female adult longevity (day)	16	34.25 ± 2.53 abc	22	38.05 ± 0.83 a	19	28.32 ± 1.65 c	23	34.87 ± 0.84 b
Male adult longevity (day)	16	37.63 ± 0.78 a	23	35.26 ± 1.77 ab	12	32.42 ± 1.24 b	23	33.70 ± 1.52 b
Adult preoviposition period (APOP) (day)	16	2.06 ± 0.26 b	22	3.86 ± 0.34 a	19	1.32 ± 0.19 c	23	1.35 ± 0.10 c
Total preoviposition period (TPOP) (day)	16	20.25 ± 0.41 b	22	22.41 ± 0.44 a	19	19.11 ± 0.33 c	23	20.87 ± 0.31 b
Fecundity (F) (eggs/female)	16	179.50 ± 15.83 b	22	278.55 ± 10.87 a	19	117.05 ± 8.66 c	23	270.65 ± 12.20 a

SEs were estimated with 100,000 bootstraps. Different letters in the same row represent significant differences with a paired bootstrap test (*p* < 0.05).

**Table 4:** Population parameters of *Chaetodactylus hirashimai*.

Population Parameter	Feed Treatment			
	Cherry Pollen	Cherry Bee Bread	Apple Pollen	Apple Bee Bread
*r* (day^−1^) intrinsic rate of increase	0.14 ± 0.01 a	0.15 ± 0.01 a	0.14 ± 0.01 a	0.15 ± 0.01 a
*λ* (day^−1^) finite rate of increase	1.15 ± 0.01 a	1.16 ± 0.01 a	1.15 ± 0.01 a	1.16 ± 0.01 a
*R*_0_ (offspring/individual) net reproductive rate	49.52 ± 11.40 b	120.16 ± 19.87 a	39.71 ± 7.93 b	115.28 ± 18.90 a
*T* (day) mean generation time	28.68 ± 0.46 c	32.96 ± 0.48 a	26.32 ± 0.36 d	31.57 ± 0.49 b

SEs were estimated with 100,000 bootstraps. Different letters in the same row represent significant differences with a paired bootstrap test (*p* < 0.05).

## Data Availability

The datasets generated during the current study are available from the corresponding author on reasonable request.
